# Characterization and performance of aluminum (III)-complex as an active material for potentiometric sensor

**DOI:** 10.1038/s41598-026-66624-9

**Published:** 2026-08-24

**Authors:** Khadega Hassan Fawzy, Mohamed A. Zayed, Eman Yossri Frag

**Affiliations:** Chemistry Department, Faculty of Science, Cairo University, Giza, 12613 Egypt

**Keywords:** Al(III) selective sensor, Modified carbon paste electrode, Flubendazole-Al(III) complex, Al(III) quantification, Applications, Real samples, Chemistry, Environmental sciences, Materials science

## Abstract

Aluminum is the third most abundant element in the Earth’s crust. It becomes more soluble and available when the soil pH falls below 5. Consequently, elevated aluminum concentrations can severely inhibit plant root growth, causing stunted roots and a reduced ability to absorb water and nutrients. Therefore, monitoring aluminum levels in soil is important, which has led to significant interest in developing sensors for its detection. Al(III)-flubendazole complex (Al-FLU) was synthesized for the first time and evaluated as a potential electroactive sensing material. This complex was incorporated into a novel, well-defined chemically modified carbon paste electrode (CPE). Tricresyl phosphate (TCP) was selected from three tested plasticizers and used as a solvent mediator in the paste matrix, complementing the Al-FLU complex. This plasticizer improved the electrode’s mechanical stability and analytical performance. With an optimal composition, the resulting electrode achieved the intended Nernstian slope of 20.0 ± 0.683 mV per decade for trivalent cations over the concentration range 1.0 × 10⁻^7^ to 1.0 × 10⁻^1^ mol L^−1^, with a detection limit of 1.0 × 10⁻^7^ mole L^−1^ and a response time of about 5.0 s. The electrode demonstrated good selectivity, sensitivity, reversibility, repeatability, and reproducibility. Its potential response remained constant across the pH range of 3.4–5.5 at room temperature (25 ± 1 °C). The thermal stability of the electrode was also evaluated between 10 and 60 °C. Therefore, this optimally formulated electrode can be used to establish a simple direct potentiometric analytical method for the systematic quantification of Al(III) in both pure solutions and various real samples.

**Supplementary Information:**

The online version contains supplementary material available at 10.1038/s41598-026-66624-9.

## Introduction

For analytical chemists, developing a new quantitative method is a key zone. Determining the metals that are important for industry and have an impact on human health has therefore been the subject of intensive research, with the goal of developing methods that are sensitive, straightforward, accurate, quick, and affordable. Among these metals, Aluminum needs precise control in the environment. Aluminum (Al) is an element present in the boron group in periodic Table. It is one of the most important heavy metals, and the second most abundant metal after Iron. After silicon and oxygen, aluminum is regarded as the element that is most naturally abundant in the crust of the planet^1,2^. Approximately 8% of all mineral components are made up of it because of its strong solubility in acid environments. Its specific weight 2.7 g cm^−3^, so it is regarded as light and strong. While aluminum’s natural state is stable and doesn’t interact with biological systems but combined with other elements; so, it is incredible. The useful physicochemical properties of aluminum include its amphoteric and the ability to transform, most commonly oxygen, silicon, and fluorine^3^, forming highly soluble forms in acid environment absorbed by living systems^4^. Thus, manufacture every aspect of our daily lives involves the use of aluminum, i.e., from water purification, food processing, cosmetics, vaccinations, medicines, to even manufacture of packaging, clothing, building construction, machinery, electronics, and telecommunication. Hence, aluminum is considered as the most widely used metal after iron. There are several studies focused on aluminum in different research fields. Al can be considered as double-edged weapon due to its both useful and deleterious impacts on our life, and this will be clearly observable from the following mentioned points. Al is found in water, air, soil, animal tissues, and plants in different forms. Anthropogenic activities cause its release into the environment, such as industrial uses, mining, and production of Al metal and its compounds. Living systems need Al in very trace amounts but high levels of Al resulting from prolonged Al intake has hazardous effects on the living systems^5^. For several years, aluminum was not considered as a dangerous metal for humans but in 1965 research on animals raised the possibility that aluminum and Alzheimer’s disease AD (dementia) were related^6^. Also, Al plays a significant role in diseases of osteomalacia, dialysis, anemia,^7^ and the pathology of Parkinson’s disease^8^. Thus, in addition to its known role in the aetiopathogenesis of some blood and bone illnesses, aluminum is also a potential neurotoxic. In addition, inhalation of high amounts of Al causes decreasing in the performance of certain tests that assess respiratory system functions^9^, among other consequences including occupational asthma^10^. Therefore, high levels of Al in our body put us at risk of deleterious effects on the brain, nerve, renal and respiratory systems. Furthermore, elevated Al levels adversely influence fish, algae, and crops^11^.

0.1 to 0.3% is the estimated oral bioavailability of Al from diet and water, depending on urine elimination and daily Al intake. More than 95% of Al is excreted via the kidney, according to the results of a few trials using a restricted diet and tea^12^. The Agency for Toxic Substances and Disease Registry (ATSDR) states that 2.0 mg/kg/day is the minimal risk level (MRL) for aluminum^13^. However, Al(III) salts and its reactive species are widely utilized as a flocculating agent in potable water treatment. Food products, soft drinks units, food contain aluminum in different amounts from 1 mg/kg to as high as multiple of 100 mg/kg (without or with food additives). Also, it is the basic component of practically every food item, including lentils, cereals, tea, fruits, spices, vegetables, and pharmaceuticals^12^. Furthermore, in medicine Al(III) compounds have been safely utilized in the treatment of peptic ulcers, gastric hyperacidity(antacids). Also, Al is used as antiperspirants, vaccine adjuvant, buffered aspirin and first aid antiseptics and antibiotic^14^. All these findings cause alarming concern of Al(III) in public health owing to its widespread use and its toxic nature. Thus, there is respective attractive for Al(III) monitoring at traces amounts. For Al abundant occurrence, there are several methods that were reported for direct determination of Al(III); which have various levels of sophistication and accuracy. These include inductively coupled plasma optical emission spectrometry (ICP–AES)^15^, electrothermal atomic absorption spectrometry^16^, inductively coupled plasma mass spectrometry (ICP-MS)^17^, spectrophotometric methods^18–22^, flow injection (FI)^23^, graphite furnace atomic absorption spectrometry (GFAAS)^24^, spectrofluorometry^25^, flame atomic absorption spectrometry^26^, HPLC^27^. Most of these techniques require sophisticated instruments and time-consuming sample preparation protocols**.** Electrochemical methods are based on directly tracking the concentration of an analyte ion in a solution and then monitoring the electrical current at the surface of an electrochemical sensor. This process converts electrochemical information into a signal that can be used analytically to determine the analyte concentration, making electrochemical methods a powerful and versatile analytical tool^28^. Electroanalytical procedures offer many more favored features rather than the earlier mentioned techniques such as specificity for a certain oxidation state of an element, applying a relatively inexpensive instrumentation. Furthermore, rather than offering information on chemical species concentrations, they offer information about activities. Moreover, the potentiometric measurements based on ion selective electrodes (ISEs) do not consume the analyte. So, they are favored to quantify lower concentration of analyte owing to their elevated sensitivity. Their procedures are controlled by the properties of the investigated electrode surface membrane; so, it is an excellent method for the determination of Al(III) quantity in different water samples including: square wave voltammetry^29^, cyclic voltammetry^30^, differential pulse voltammetry^31^ and potentiometric methods based on ISEs^32–38^. ISEs are typically created using a membrane selective to ion and an electronic transduction mechanism to transform the chemical potential difference between a reference electrode and an indicator electrode in solution, while the current is maintained at zero is converted to electrical potential^39^. Their selectivity is achieved by doping the membrane with ion-selective complexing agents or by modifying the material structure. Hence, ISEs have several advantages including linear dynamic response range as well as a low detection limit of the analyte, no consumption of analyte, short response time, non-destructive, environment friendly. The simple instrumentation and sample’s turbidity or color has no effect on the response^40^. Nowadays, ISEs are successfully applied in pharmaceutical, clinical, environmental, monitoring process field and electrochemical analysis. Recently, Carbon paste electrodes (CPEs); which are a type of potentiometric ISEs; have attracted attention as ion selective electrodes for more widely successful applying over than other ISEs in analytical procedures, mainly due to their easiness of modification of compositions^41^, incorporating nanostructures to enhance the electrochemical transformation of electroactive molecules, compatibility with various types of modifiers as different ligands based on the application can be easily mixed during the preparation of paste, moreover, CPEs are being non-destroyable (mechanically strong) due to their solid chemical inertness nature providing an easily improved renewable surface. So, they time-saving, fast stable response^42^ and low Ohmic resistance^43^, low background current interferences during analysis^44^, use of less expensive materials^45^, do not require internal solutions. Hence, the strategy is the chemical modification of electrode surfaces for enhancing the analytical performance of traditional electrode materials for certain applications in different fields, especially electroanalysis and sensors. Al(III)-Flubendazole was shown to be very suitable for the development of a novel modified electrode.

To the best of our knowledge, this is the first time the Al(III)-Flubendazole complex has been synthesized and characterized by mass spectrometry, elemental analysis, and DFT calculations, and used as the electroactive material to build a new carbon paste electrode (CPE) selective for Al(III) ions. The potentiometric response of the CPE for aluminum determination in real samples was measured, and the electrode performance was optimized.

## Experimental

### Reagents and solutions

All the reagents and chemicals used were of analytical-reagent grade purity unless otherwise stated. Tricresylphosphate (TCP) and extra pure graphite fine powder (particle size < 50 μm), and acetone were supplied from Aldrich. Dibutylphthalate (DBP) was supplied by BDH Industries Ltd., while O-Nitrophenyloctylether (o-NPOE) was supplied by Fluka, Al_2_ (SO_4_)_3_.16H_2_O, NiCl_2_.6H_2_O and MnCO_3_, were supplied from BDH chemicals Ltd Poole England; CrCl_3_.6H_2_O from laboratory chemicals; SrCl_2_.6H_2_O from Laboratory Rasayan; MgCO_3_ from Fischer scientific. Pb (CH_3_COO)_2_. 3H_2_O purchased from Elmadina For chemicals, CaCL_2_ from Adwic. BaCl_2_ 0.2H_2_O, KCl and NaCl from S.B.O. (Scientific Bismarco Office) CoCl_2_.6H_2_O and CuCl_2_.2H_2_O were supplied by El-Nasr Company. H_2_SO_4_ (Conc.), NaOH and HCl (Conc.) from ADWIC Pharmaceutical. FLU was gifted from the Egyptian National Organization for drug control and research (Nodcar).

Fresh standard stock solution (1.0 × 10^−1^ mol L^−1^) of Al (III) was prepared by dissolving the accurate weighed amount in 100 mL distilled water to get the required concentration. Other dilute solutions (5.0 × 10^−2^–1.0 × 10^−9^ mol L^−1^) were prepared from stock solution by serial dilution. All solutions must be kept out of the light by being stored in dark-colored quick-fit bottles during the whole work.

0.1488 g of air-dried soil sample was weighed and digested as mentioned by Marion LeRoy Jackson^46^, then subjected to Al(III) quantification by the developed electrode and ICP. Sandy soil was supplied from Agriculture Station at Ismailia governorate, Agriculture Research Center (ARC), Egypt.

Food products were collected from local market of Giza city, Egypt and digested, then subjected to Al(III) quantification by the developed electrode and ICP.

### Preparation of Al-FLU complex (ionophore)

The Al(III)–FLU complex ionophore was synthesized by reacting Al(III) with flubendazole. This was achieved by mixing 0.630 g of Al_2_(SO_4_)_3_·16H₂O with an appropriate amount of flubendazole in a minimal volume of ethanol, with continuous stirring for about two hours at room temperature until a complex formed. The solution was then allowed to evaporate gradually at room temperature. The resulting complex was filtered, washed several times with small volumes of solvent, and dried over anhydrous calcium chloride. Mass and elemental analysis confirmed the composition of the prepared solid complex.

### Preparation of chemically modified electrode

Ion-sensitive CPEs were prepared following previously published procedures^47,48^. The electrode paste was formed by intensive homogenization, achieved by mixing and grinding the Al-(FLU)_2_ complex (0–40 mg) with 500 mg of high-purity graphite powder and 0.2 mL of a plasticizer (o-NPOE, DBP, or TCP) for approximately 30 min, until complete homogenization. The resulting paste was packed into the electrode body, and a wet filter paper was used to smooth the carbon paste until it became glossy^49^.

### Apparatus and emf measurements

To gauge the selectivity of the Flubendazole-Al(III) complex as an ionophore in the proposed paste, preliminary experiments were conducted prior to optimization. The proposed electrode was prepared and calibrated by immersion, in conjunction with a saturated calomel reference electrode, in a cell containing 5.0 mL aliquots of various metal ions separately, ranging from 1.0 × 10⁻^8^ to 1.0 × 10⁻^1^ mol L⁻^1^. The calibration of the developed electrode was established by plotting the recorded potential difference against − log[M^n+^]. A fresh paste was used for each calibration.”

## Results and discussion

### Physicochemical characteristics of Al(III)-flubendazole complex

**Fig. 1:**
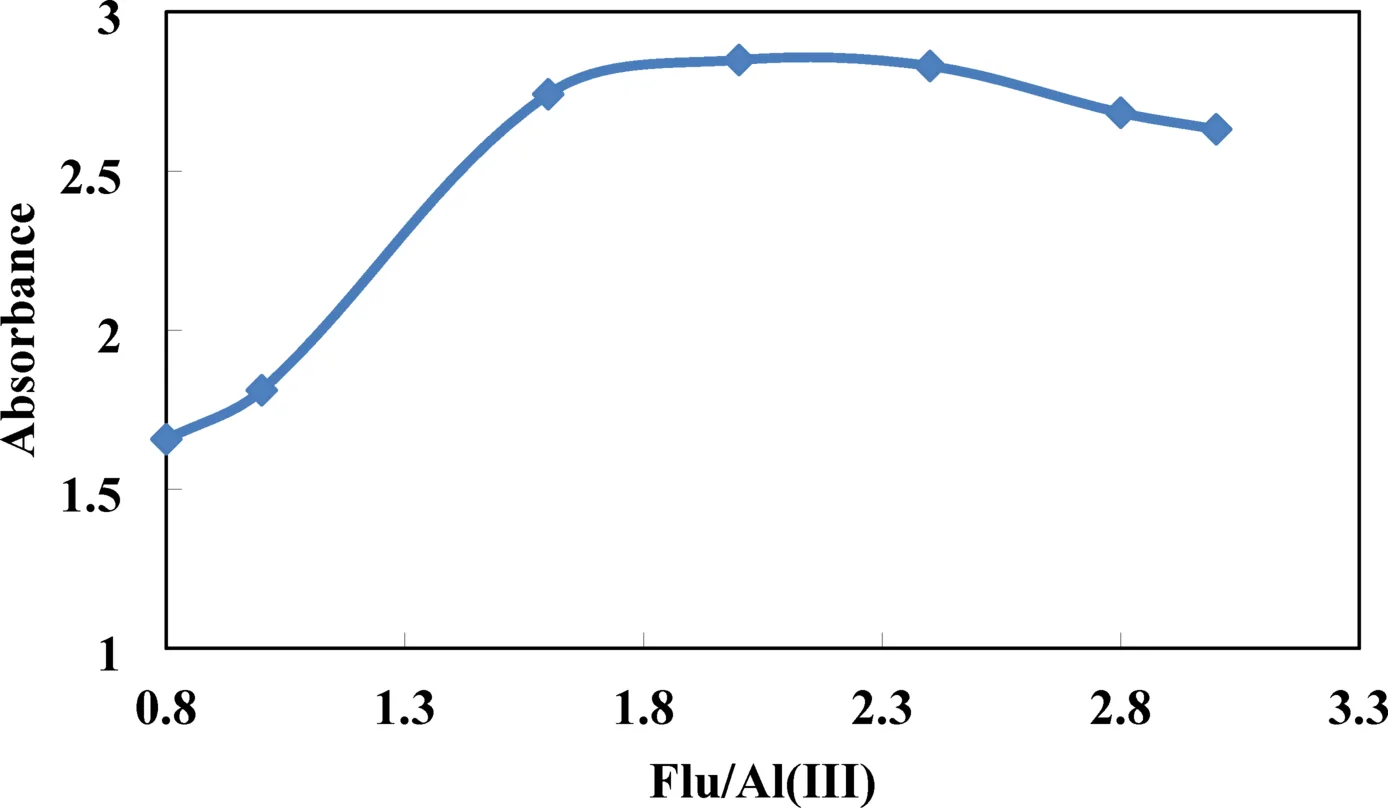
Molar ratio method for Al(III) with flubendazole at 314 nm.

The Al(III)-flubendazole complex was characterized by elemental analysis: molecular formula [(C_16_H_12_FN_3_O_3_)_2_⋅ Al ]1.5 SO_4_, Mol. Wt.: 797.54 g mol⁻^1^; color: white; %C, 48.5 (48.8); %H, 3.1 (4.7); %N, 10.5 (10.6). A good agreement was observed between the found and calculated values. The stoichiometric ratio between Al(III) and flubendazole was investigated using the molar ratio method (at 314 nm, Supplementary Material Fig. S1); as depicted in Fig. 1, the ratio of Al(III) to flubendazole was found to be 1:2.

The molar conductance of the Al(III)–flubendazole complex in DMF was found to be 90 Ω⁻^1^ cm^2^ mol⁻^1^, indicating its electrolytic nature. This suggests that the sulfate anion is present as an outer-sphere counter ion rather than being directly coordinated to the Al(III) center. In addition, the FT-IR spectrum of the Al(III) complex as shown in Supplementary Material Fig. S2 exhibited characteristic absorption bands at 1111.86 and 591.48 cm⁻^1^, which are assigned to the vibrational modes of the sulfate ion, respectively, while the band at 972.12 cm⁻^1^ can be attributed to the vibration of SO_4_^2^⁻^50^. These bands confirm the presence of sulfate in the isolated complex. The absence of clear spectral evidence for sulfate coordination suggests that the sulfate ions most likely act as counter anions, whereas coordination to the Al(III) center occurs through the benzimidazole nitrogen and the carbamate carbonyl oxygen atoms.

**Fig. 2:**
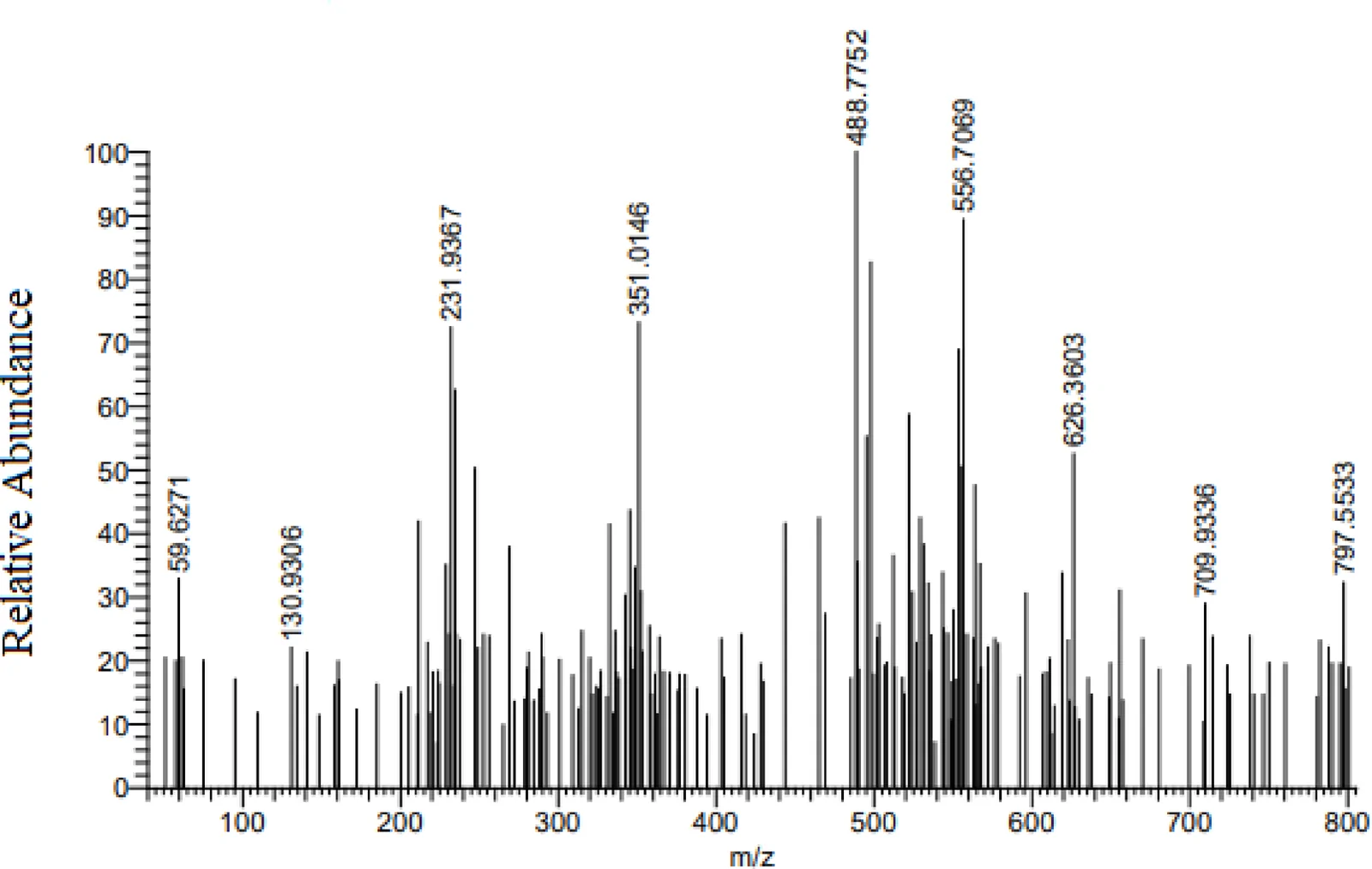
Mass spectra of Al(III)-FLU complex.

*Mass Spectral Studies*: The mass spectrum of a compound displays signal intensities at various m/z values. Because no two compounds exhibit identical mass spectra, this profile serves as a highly distinguishing feature for identification. Although other fragmentation peaks were present, this study focused solely on the molecular ion peaks of the Al(III)-FLU complex at m/z 797.55, corresponding to [(C_16_H_12_FN_3_O_3_)_2_⋅ Al]1.5 SO_4_ (Mol. Wt. = 797.54 g mol⁻^1^), and at m/z 387.75, corresponding to [(C_16_H_12_FN_3_O_3_)⋅ Al]1.5 SO_4_ (Mol. Wt. = 388.30 g mol⁻^1^), while the peak at m/z 313.066 corresponds to the free flubendazole ligand (C_16_H_12_FN_3_O_3_) (Mol. Wt. = 313.28 g mol⁻^1^), as illustrated in Fig. 2.

**Fig. 3:**
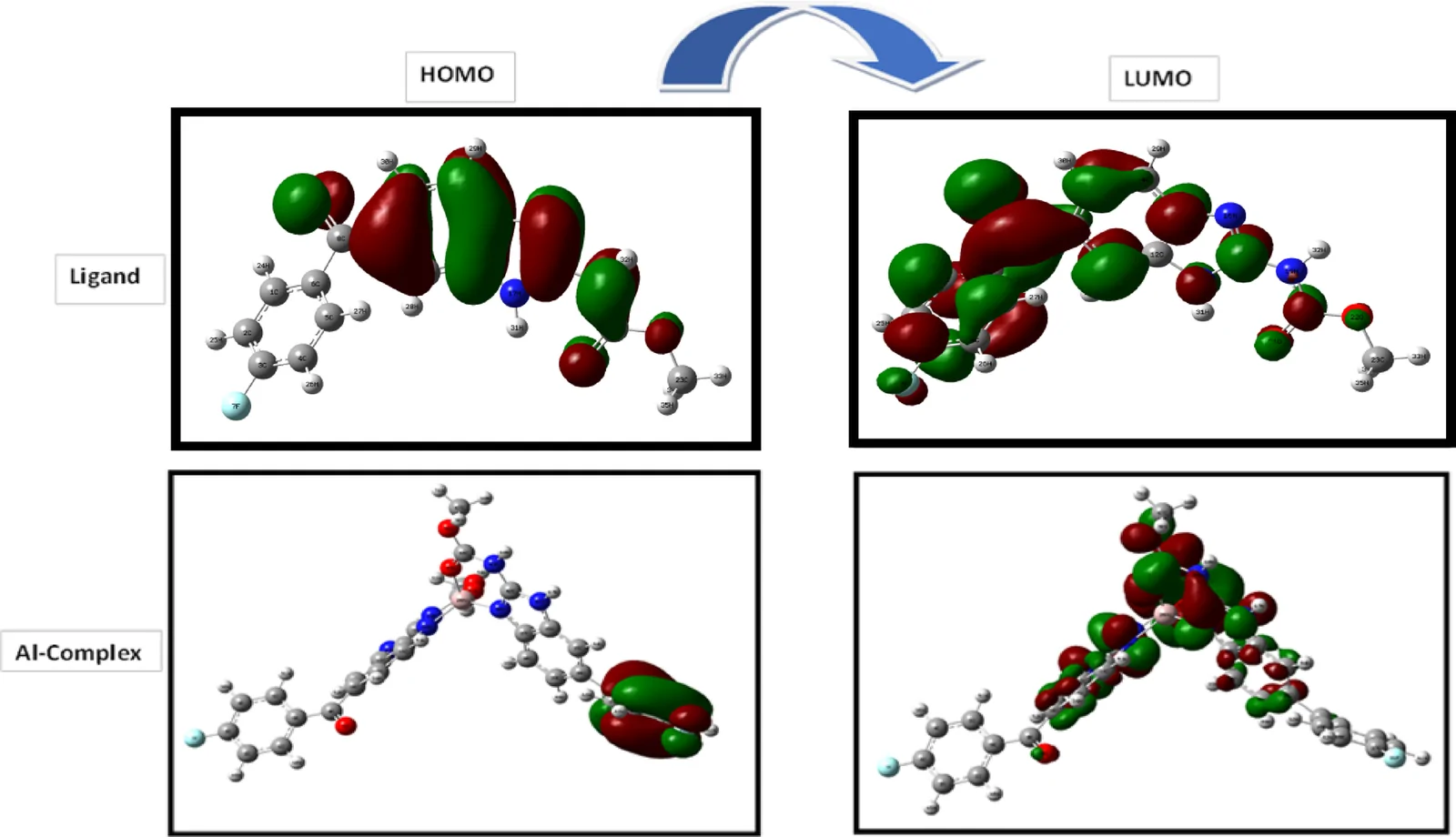
Molecular graphs of FLU and its aluminum complex.

**Fig. 4:**
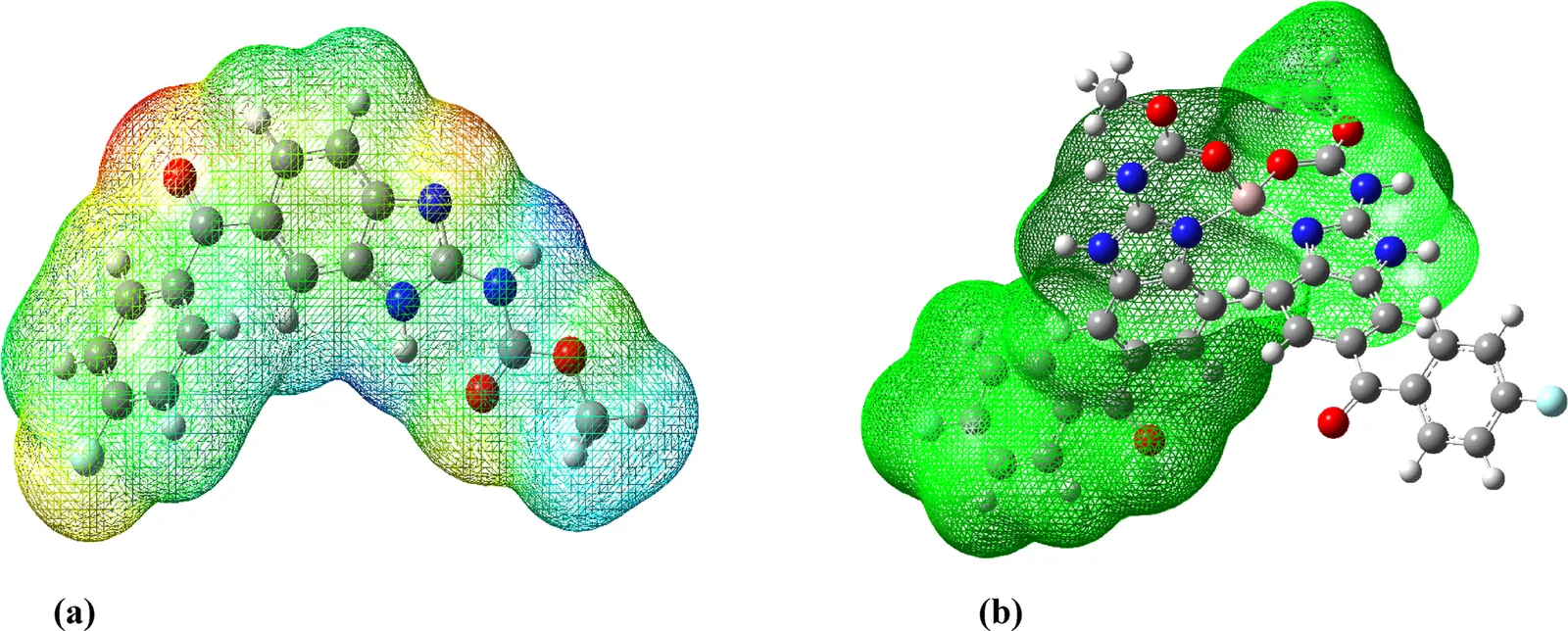
MEP of (**a**) flubendazole and (**b**) Al(III)-FLU complex.

*Geometry Optimization*: Density functional theory (DFT/B3LYP) calculations with the LANL2DZ basis set were performed using GaussView 5.0.8^51,52^ to optimize the geometries of flubendazole and its aluminum complex, thereby confirming the reported characterization data (Supplementary Material Fig. S3). The resulting optimization energy, energy gap, hardness (η), and dipole moment values are listed in Table 1. Flubendazole exhibits geometric changes upon complexation with aluminum ions, where most bond lengths increase, as shown in Supplementary Material Table 1. The energies for the highest occupied molecular orbital (HOMO) and lowest unoccupied molecular orbital (LUMO) are presented in Table 1 and depicted in Fig. 3. The calculated quantum chemical parameters indicate that complexation with Al(III) significantly modifies the electronic properties of flubendazole (Table 1). The HOMO–LUMO energy gap decreases markedly from 4.339 eV for the free ligand to 1.442 eV for the Al(III) complex, indicating enhanced chemical reactivity. This is further supported by the decrease in global hardness (η) from 2.169 to 0.721 eV and the corresponding increase in softness (S) from 0.230 to 0.693 eV⁻^1^. Moreover, the dipole moment increases substantially from 6.886 to 29.911 Debye, reflecting the increased molecular polarity induced by coordination with Al(III). As shown in Fig. 3, the HOMO of the free ligand is mainly localized over the benzimidazole moiety and the conjugated framework, whereas the LUMO is distributed over most of the molecular skeleton. After complexation, the HOMO becomes mainly localized around the Al(III) coordination site and the donor atoms, while the LUMO is concentrated predominantly on the fluorophenyl ring. This redistribution of the frontier molecular orbitals suggests enhanced intramolecular charge transfer upon excitation, which is consistent with the reduced energy gap and increased electronic reactivity of the complex (Fig. 4).

**Table 1:** Ground state properties of FLU and its Al complex using B3LYP/6-311G and B3LYP/LANL2DZ respectively.

Parameter	FLU	Al-complex
E_T_, Hartree	− 1106.698	− 2214.352
E_HOMO_, eV	− 6.451	− 12.592
E_LUMO_, eV	− 2.112	− 11.149
ΔE, eV	4.339	1.442
I = - E_HOMO_, eV	6.451	12.592
A = - E_LUMO_, eV	2.112	11.149
χ, eV	1.974	16.458
η, eV	2.169	0.721
S, eV^−1^	0.230	0.693
µ, eV	− 4.282	− 11.870
Dipole moment (Debye)	6.886	29.911

### Optimization of the electrode membrane composition

The critical factor in developing a functionalized ion-selective electrode (ISE) is the ionophore itself. Consequently, the current work explores several approaches to develop a highly selective ionophore. Changing the relative proportions of the carbon paste electrode (CPE) ingredients (ionophore and plasticizer) results in detectable changes in the potentiometric transduction slope, sensitivity, and linearity. This underscores the significant dependence of the analytical response characteristics of the CPE on the type and amount of the ionophore, as well as the nature of the plasticizer^53^. The ionophore (electroactive material) is the component of the CPE responsible for electrode selectivity. Previous studies^54^ indicate that ionophores, in conjunction with lipophilic ionic sites, govern the response of the ISE.

Incorporating the Al(III)-FLU complex (ionophore) into the paste directly affects the sensitivity of the potentiometric CPE. Consequently, the initial stage of the experimental work aimed to assess the selectivity of the designed electrode. CPEs modified with varying proportions of the Al(III)-FLU complex were evaluated against several metal ions separately as depicted in supplementary material Figs. S4 and S5. Among all tested cations, Al(III) exhibited the optimal characteristic response, demonstrating a Nernstian potential response over a wide linear range. This confirms that the synthesized Al(III)-flubendazole complex acts as a highly selective ionophore for Al(III) ions, providing a functionalized CPE for the quantitative determination of Al(III) ions.

Six CPEs were prepared with different mass ratios of the Al(III)-FLU complex, graphite powder, and TCP plasticizer to evaluate their performance in the potentiometric quantification of aluminum ions by constructing their calibration plots. The corresponding linear ranges, slopes, and correlation coefficients are presented in Table 2. As shown in Table 3, the sensor containing 2.67% (wt/wt) Al(III)-FLU, 30.54% (wt/wt) TCP, and 66.79% (wt/wt) graphite powder (sensor IV) exhibited the optimal composition, demonstrating a trivalent Nernstian slope of 20.0 ± 0.683 mV decade⁻^1^ within a linear concentration range of 1.0 × 10⁻^7^ to 1.0 × 10⁻^1^ mol L⁻^1^ and a detection limit of 1.0 × 10⁻^7^ mol L⁻^1^. In contrast, a lower content of the Al(III)-FLU complex resulted in lower slope values, whereas a higher content caused oversaturation and inadequate performance due to steric hindrance at the interface. The unmodified paste showed no significant response under the optimum conditions.

**Table 2:** Effect of Al(III)-FLU complex content and type of plasticizers on the CPE performance for Al (III) ions potentiometric calibration.

Sensor no	Paste composition % wt/wt				Linear range (mol L^−1^)	Slope, mv decade^−1^	Correlation coefficient (r^2^)
	Graphite powder	Ionophore (AL-FLU)	plasticizer	Plasticizer type			
I	68.62	0.00	31.37	TCP	1.0 × 10^−5^–1.0 × 10^−1^	13.38 ± 0.44	0.9209
II	68.16	0.68	31.16	TCP	1.0 × 10^−7^–1.0 × 10^−1^	15.67 ± 0.85	0.9943
III	67.69	1.354	30.95	TCP	1.0 × 10^−7^–1.0 × 10^−1^	17.70 ± 0.75	0.9986
IV	66.79	2.67	30.54	TCP	1.0 × 10^−7^–1.0 × 10^−1^	20.00 ± 0.68	0.9949
V	65.91	3.95	30.13	TCP	1.0 × 10^−7^–1.0 × 10^−1^	21.39 ± 0.76	0.9907
VI	65.05	5.20	29.74	TCP	1.0 × 10^−7^–1.0 × 10^−1^	25.70 ± 0.89	0.9752
VII	68.68	2.75	28.57	o-NPOE	1.0 × 10^−7^–1.0 × 10^−1^	30.7 ± 0.74	0.9916
VIII	68.62	2.745	28.63	DBP	1.0 × 10^−6^–1.0 × 10^−1^	28.72 ± 1.05	0.9712

**Table 3:** Potentiometric selectivity coefficients of the developed sensor using SSM Linear concentration range, slope values, and correlation coefficient of calibration plots of some interfering ions.

Interfering Species	Slope mV decade^−1^	correlation coefficient, r^2^	Linear concentration range, mol L^−1^	Log K^pot^_Al_^3+^_, B_
Cu(II)	15.4	0.8732	1.0 × 10^−5^–1.0 × 10^−2^	1.45
Cr(III)	16.43	0.9925	1.0 × 10^−4^–1.0 × 10^−1^	− 1.85
Ni(II)	23.32	0.988	1.0 × 10^−5^–1.0 × 10^−1^	− 4.35
Co(II)	16.35	0.9708	1.0 × 10^−6^–1.0 × 10^−1^	− 4.1
Ca(II)	17.74	0.9873	1.0 × 10^−5^–1.0 × 10^−1^	− 0.20
Mn(II)	14.80	0.9826	1.0 × 10^−6^–1.0 × 10^−2^	− 1.70
Pb(II)	18.6	0.8688	1.0 × 10^−6^–1.0 × 10^−3^	− 5.25
Sr(II)	23.0	0.9975	1.0 × 10^−4^–1.0 × 10^−2^	− 0.70
Mg(II)	21.5	0.9857	1.0 × 10^−5^–1.0 × 10^−2^	− 1.35
Ba(II)	23.2	0.9861	1.0 × 10^−5^–1.0 × 10^−2^	− 1.20
K(I)	26.2	0.9856	1.0 × 10^−6^–1.0 × 10^−1^	− 1.25
Na(I)	22.6	0.9385	1.0 × 10^−6^–1.0 × 10^−1^	− 1.15

*The nature and proportion of the plasticizer* must be optimized to eliminate electrical asymmetry and enhance the electrochemical activity of the fabricated paste. Plasticizers are non-conducting organic mediators that provide suitable viscoelastic properties to the paste while reinforcing its mechanical stability and resistance to erosion in solutions. Furthermore, binding between particles expands the active surface area of the carbon paste^55^. Consequently, plasticizers serve dual functions: facilitating homogeneous solubilization of the components and optimizing the ionophore’s distribution constant. Additionally, the plasticizer influences the detection limit, paste selectivity, and ion extraction into the paste phase^56^.

To determine the most suitable plasticizer for constructing the proposed electrode, several carbon pastes were prepared using different plasticizers, namely *o*-NPOE, DBP, and TCP. Their effects were evaluated, and the results obtained are presented in Table 2. The electrode plasticized with TCP (sensor IV) yielded the best Nernstian slope, reproducibility, and stability. This optimal performance is attributed to minimized electrical asymmetry, enhanced flexibility, and improved durability, which altogether produce a more homogenous paste. The performance of the CPE relies on the selective extraction of the target ion, which establishes the electrochemical phase boundary potential via thermodynamic equilibria at the sample/electrode interface. The super-Nernstian slopes observed for membranes plasticized with o-NPOE and DBP are attributed to deviations from ideal Nernstian behavior arising from changes in the physicochemical properties of the membrane. Both plasticizers increase membrane flexibility, reduce electrical resistance, and enhance the mobility of the Al(III)–ionophore complex within the membrane. Owing to its high dielectric constant, o-NPOE promotes the partitioning and transport of ionic species more effectively than less polar plasticizers, whereas DBP improves membrane fluidity and facilitates ion diffusion. These properties can accelerate interfacial ion-exchange kinetics and alter the membrane/solution equilibrium, particularly at higher analyte activities. Consequently, slight deviations from the theoretical Nernstian response, including super-Nernstian slopes, may occur because of nonequilibrium interfacial processes, transient ion fluxes, or membrane conditioning effects rather than enhanced complexation by the plasticizer itself. Similar deviations from ideal Nernstian behavior have been reported for polymeric ion-selective electrodes and are generally attributed to membrane transport and interfacial equilibrium phenomena rather than to the intrinsic recognition properties of the plasticizer.

### Electrode performance

The electrochemical performance parameters of the developed electrode (sensor IV) were comprehensively evaluated in accordance with the IUPAC recommendations^57^ under various experimental conditions. This systematic assessment was conducted to verify its analytical suitability and optimal properties.

*The pH response profile* of the proposed electrode for a 1.0 × 10⁻^3^ mol L⁻^1^ Al(III) solution was investigated over the pH range of 1.9–11.0 to determine its operational pH range. The experimental data revealed that the potential response remains constant within the pH range of 3.4–5.5, as shown in Fig. S6 (Supplementary Material). Therefore, the potential response of the proposed electrode is pH-independent within this specific range. At pH > 5.5, the potential response decreases due to the precipitation of Al(OH)_3_ or the formation of soluble hydroxy complexes, which lowers the free Al(III) ion concentration in the solution. Conversely, at acidic pH values (pH < 3.4), the potential response increases somewhat erratically owing to the electrode’s sensitivity to H⁺ activities.

**Fig. 5:**
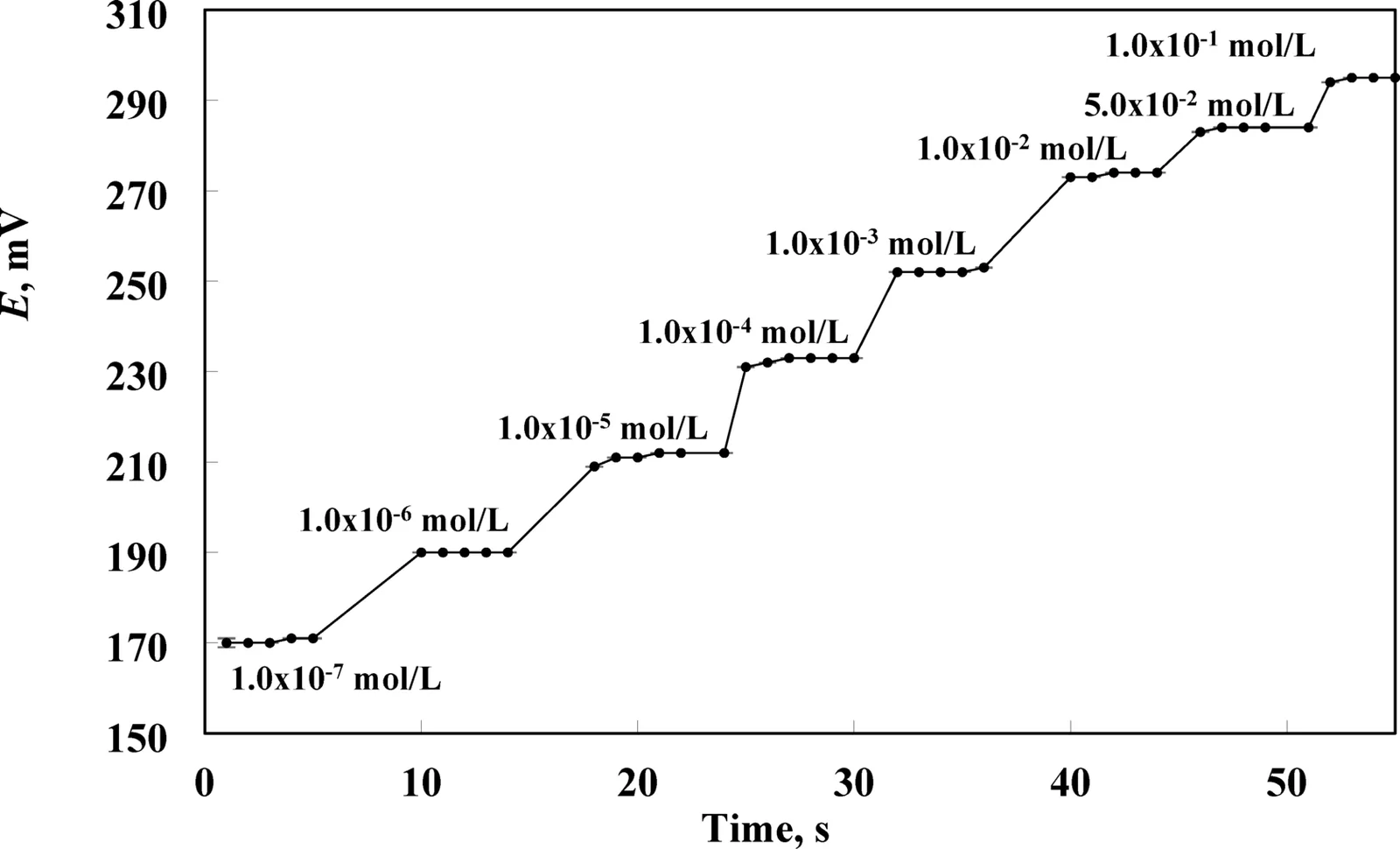
Sensor IV response time in conjugation with saturated reference calomel electrode.

In accordance with IUPAC recommendations, *the response time of the CPE* is defined as the time elapsed from its immersion, along with the reference electrode, into the analyte solution until the equilibrium potential remains constant^58^. In the present work, the response time was evaluated by measuring the time required for the CPE to achieve 95% of its steady-state potential value (within a variation of ± 1 mV) following successive immersion in a series of aluminum ion solutions, each featuring a tenfold increase in concentration from 1.0 × 10⁻^7^ to 1.0 × 10⁻^1^ mol L⁻^1^ of Al^3^⁺^59^; the results are presented in Fig. 5. The CPE attained a stable equilibrium potential within a brief duration of approximately 5.0 s. This rapid response reflects the fast kinetic exchange of aluminum ions with the ionophore at the electrode/solution interface, as well as the optimal ionophore content and the appropriate choice of a plasticizer with a high dielectric constant.

*The thermal stability of the fabricated electrode *was investigated by recording calibration curves at various solution temperatures (10–60 °C) to evaluate the thermal effect on the sensor’s performance. To determine the isothermal coefficient (dE°/dt), the standard electrode potentials (E°) obtained at different temperatures were plotted against (t − 25), where (t) represents the experimental temperature in degrees Celsius. According to Antropov’s equation^60^, a linear relationship was obtained, where the slope represents the isothermal coefficient of the electrode. E^o^cell = E^o^cell(25°C) + (dE^o^cell/dT) (t − 25).

The calculated coefficient was 1.778 × 10⁻^3^ V/°C. This low value indicates that the developed electrode is thermally stable and operational at elevated temperatures up to 60 °C without any detectable deviation from Nernstian behavior, while maintaining robust mechanical stability.

*The interference study* is a critical parameter for evaluating the selectivity of CPEs, as it determines the preference of the electrode membrane for aluminum ions over various interfering species, thereby establishing its applicability in real sample analysis. In this study, the potentiometric selectivity coefficients (log K^pot^_Al3+,B_) of the CPE were determined using the Separate Solution Method (SSM)^61,62^ according to the following equation:

$$\log \mathrm{K}^{{\mathrm{pot}}_{\mathrm{Al}^{3+,_{\mathrm{B}}}}} = \left[ {\left( {\mathrm{E}_{\mathrm{B}}{-}{\mathrm{E}}_{{\mathrm{Al}^{{3 + }}}}} \right)/\mathrm{S}} \right] + \left( {1{-}\left( {\mathrm{Z}_{{\mathrm{Al}^{{3 + }}}} /\mathrm{Z}_{\mathrm{B}} } \right)} \right)\log\mathrm{a}_{{\mathrm{Al}^{{3 + }}} }$$

where E_Al3+_ and E_B_ represent the potentiometric responses of the primary ion and the interfering ion, respectively. The variables a_Al_^3+^ and a_B_ denote the activities of the aluminum ion and the interfering species, where a_Al_^3+^ = a_B_ = 1.0 × 10^−3^ mol L^−1^. Additionally, S is the Nernstian slope, while Z_Al_^3+^ and Z_B_ correspond to the charges of the primary and interfering ions, respectively. In the SSM approach, the potentials of the Al(III) and interfering ion solutions were measured separately. Based on the selectivity coefficients listed in Table 3, aluminum ions were highly preferred over the interfering species. These results were further verified by recording calibration curves for all interfering ions to evaluate their linear concentration ranges, slope values, and correlation coefficients. Mechanistically, Al(III) is a hard Lewis acid with a high charge density, enabling it to interact effectively with the oxygen and nitrogen donor atoms of flubendazole. The strong electrostatic interactions and the covalent character of the Al–O bonds contribute significantly to the stability of the complex. The pre-formed Al(III)–flubendazole complex is incorporated as a highly specific ionophore within the hydrophobic carbon paste matrix, optimized by a suitable plasticizer such as TCP. The selectivity of CPE modified with the Al(III)- flubendazole complex towards Al(III) ions is primarily due to a potentiometric exchange mechanism that relies on the strong, specific, and bulky nature of the pre-formed Al(III)- flubendazole complex acting as a highly specific ionophore. When the modified CPE is immersed in an aqueous solution containing Al(III) ions, a selective ion-exchange process occurs at the electrode-solution interface. The Al(III) ions from the solution interact with the flubendazole ligands of the immobilized complex in a reversible manner. The change in concentration of the Al(III) ions in the surrounding aqueous solution generates a potential difference across the electrode interface. The electrode measures this potential change, which is proportional to the concentration of Al(III) ions in the sample, yielding a Nernstian response.

DFT calculations reveal a remarkable increase in the dipole moment upon Al^3^⁺ coordination (6.886 → 29.911 D), indicating substantial charge redistribution and increased molecular polarity. This finding, together with the marked reduction in the HOMO–LUMO energy gap and chemical hardness and the concomitant increase in softness, supports the formation of a stable and highly polarized Al^3^⁺ complex. Therefore, the excellent sensing performance toward Al^3^⁺ is attributed to the synergistic contribution of enhanced electrostatic interactions. Combined with the high charge density and strong Lewis’s acidity of Al^3^⁺, this may contribute to the preferential coordination of aluminum over mono- and divalent ions^63–67^.

### Analytical application

The fabricated Al(III) ISE was successfully utilized for the potentiometric quantification of Al(III) in soil and various foodstuff samples. The chemical composition of the soil sample is presented in Table 4. The pH of all samples was adjusted to fall within the operational pH range of the developed electrode before undergoing Al(III) analysis via the potentiometric calibration method using acetate buffer to eliminate the matrix effects due to complexing anions like, phosphates and citrates. The results obtained by the proposed electrode were in good agreement with those obtained by the ICP method, as detailed in Table 5.

**Table 4:** Composition of soil sample.

K^+^, ppm	Ca^2+^, ppm	Mg^2+^, ppm	Na^+^, ppm	Fe, ppm	Mn^2+^, ppm	Zn^2+^, ppm
1.06	1.84	0.83	0.263	1.65	0.04	0.55

**Table 5:** Determination of Al(III) ion in surface soil sample and some food stuff using the developed CPE and ICP method.

Samples	Found, ppm		t- test
	CPE	ICP	
Soil	3.885	3.5144	1.09
Fresh potato	290.04	289.9	0.41
Rice	3.97	3.430	1.62
Mushrooms	35.00	34.20	2.35

At n = 5 and 95% confidence level, the tabulated t- values is 2.571.

## Comparative studies with the previously published

The performance of the proposed carbon paste electrode was compared with previously reported potentiometric methods for Al(III) determination. Table 6 summarizes the comparative analytical parameters, including the Nernstian slope, linear dynamic range, detection limit, working pH range, and response time. As indicated in Table 6, most of the previously reported methods rely on PVC membrane ion-selective electrodes, which exhibit several drawbacks. These include the requirement of an internal reference solution that increases system impedance and response time, the leaching of electroactive components into the contacting solutions, and the inability to withstand high pressures due to the internal solution compartment. The comparative data listed in Table 6 clearly demonstrates the superiority of the proposed sensor over most of the existing methods, while remaining comparable in a few cases.

**Table 6:** Comparison between the proposed electrode and previously published methods.

Type of electrode	Modifier	Slope, mV decade^−1^	Linear range mol L^−1^	pH	Detection limit mol L^−1^	Response time, s	References
PVC	N,N′-propanediamide bis(2-salicylideneimine) (NPBS)	19.4 ± 0.3	7.9 × 10^−7^–1.0 × 10^−1^	2.5–4.0	4.6 × 10^−7^	8	^68^
PVC	salicylaldehyde salicyloyl hydrazone(SSH)	20.0 ± 0.2	9.0 × 10^−6^–1.0 × 10^−1^	2.5–4.0	7.0 × 10^−6^	9	^69^
PVC	E-N′-(2-hydroxy-3-methoxybenzylidene) benzohydrazide	19.9 ± 0.3	3.0 × 10^−7^–1.0 × 10^−2^	3.0–7.0	1.7 × 10^−7^	10	^70^
CPE		20.1 ± 0.4	1.0 × 10^−7^–1.0 × 10^−2^		5.6 × 10^−8^		
PVC	SSDA [schiff base, bis(5-sulphonate salicylaldehyde) 2,3-diaminobenzene]	15.6	1.0 × 10^−5^–1.5 × 10^−1^	3.5–4.5	5.1 × 10^−6^		^71^
PVC	Z)-2-(2-methyl benzylidene)-1-(2,4-dinitrophenyl) hydrazine (L)	20.1 ± 0.5	1.0 × 10^−6^–1.0 × 10^−1^	3.5–9.1	8.8 × 10^−7^	6	^34^
CGE			1.5 × 10^−7^ 1.0 × 10^−1^		9.2 × 10^−8^		
CWE			5.5 × 10^−7^–2.0 × 10^−1^		3.3 × 10^−7^		
PVC	a xanthone derivative, 1-hydroxy-3-methyl-9H-xanthen-9-one (HMX)	20.0 ± 0.2	1.0 × 10^−6^–1.6 × 10^−1^	3.0–8.5	6.0 × 10^−7^	5	^72^
PVC	1,10-[(methylazanediyl)bis(ethane-2,1-diyl)]bis[3-(naphthalen-1-yl)thiourea] (ACH)	17.70 ± 0.13	1.0 × 10^−6^–1.0 × 10^−2^	2.0–8.0	2.45 × 10^−7^	50	^37^
MCPE	Al (III) dicloxacillin complex	20.0	1.0 × 10^−6^–1.0 × 10^–2^	4.5–6.5	1.0 × 10^−6^	10	^73^
PVC		19.3	4.07 × 10^−7^–5.0 × 10^−5^	3.9–7.5	3.16 × 10^−7^	20	
CG		16.7	4.04 × 10^−4^–1.14 × 10^−2^	2.6–4.5	4.04 × 10^−4^	15	
CPE	Al(III)-FLU complex	20.00 ± 0.68	1.0 × 10^−7^–1.0 × 10^−1^	3.4–5.5	1.0 × 10^−7^	5	The present work

## Conclusion

A novel, simple Al(III) ion-selective carbon paste electrode (CPE) modified with the Al(III)–FLU complex was developed. The proposed electrode exhibits a Nernstian slope of 20.0 ± 0.683 mV decade⁻^1^ for trivalent cations within a linear concentration range of 1.0 × 10⁻^7^ to 1.0 × 10⁻^1^ mol L⁻^1^, achieving a detection limit of 1.0 × 10⁻^7^ mol L⁻^1^ and a rapid response time of approximately 5.0 s. The potential response remains constant over the pH range of 3.4–5.5 at room temperature (25 ± 1 °C). Under optimized composition, the CPE demonstrates high experimental utility for the successful potentiometric quantification of Al(III) ions over interfering species. It was successfully applied to analyze Al(III) in its pure form, surface soil, and various foodstuff samples, yielding reliable results comparable to those obtained by inductively coupled plasma (ICP) spectrometry, thereby validating the analytical performance of the proposed sensor.

## Supplementary Information

Below is the link to the electronic supplementary material.

Supplementary Material 1

## Data Availability

The data supporting this article has been included as part of the manuscript.
