# Supplementary material for: Characterization and performance of aluminum (III)-complex as an active material for potentiometric sensor
Source: Sci Rep. 2026 Aug 24;16:26500. doi: 10.1038/s41598-026-66624-9 (PMC13503842; doi:10.1038/s41598-026-66624-9)
Supplement: Supplementary file 1 [file 41598_2026_66624_MOESM1_ESM.docx]

| Wavelength,nm | Absorbance |
| --- | --- |
| 272 | 1.526 |
| 274 | 1.426 |
| 276 | 1.302 |
| 278 | 1.2 |
| 280 | 1.121 |
| 282 | 1.085 |
| 284 | 1.028 |
| 286 | 1.004 |
| 288 | 0.986 |
| 290 | 0.966 |
| 292 | 0.971 |
| 294 | 0.974 |
| 296 | 0.986 |
| 298 | 1.001 |
| 300 | 1.038 |
| 302 | 1.067 |
| 304 | 1.133 |
| 306 | 1.165 |
| 308 | 1.193 |
| 310 | 1.197 |
| 312 | 1.217 |
| 314 | 1.244 |
| 316 | 1.209 |
| 318 | 1.213 |
| 320 | 1.172 |
| 322 | 1.099 |
| 324 | 1.028 |
| 326 | 0.958 |
| 330 | 0.851 |
| 332 | 0.778 |
| 334 | 0.712 |
| 336 | 0.645 |
| 338 | 0.591 |
| 340 | 0.589 |
| 342 | 0.472 |
| 344 | 0.44 |
| 348 | 0.399 |
| 350 | 0.375 |
| 352 | 0.342 |
| 354 | 0.318 |
| 356 | 0.292 |
| 358 | 0.271 |
| 400 | 0.25 |
| 420 | 0.035 |
| 422 | 0.024 |
| 424 | 0.022 |
| 426 | 0.02 |
| 428 | 0.019 |
| 430 | 0.017 |
| 432 | 0.017 |
| 434 | 0.014 |
| 436 | 0.015 |
| 438 | 0.013 |

Figure 1: Absorption spectrum of flubendazole.

**Figure (2): FTIR spectra of Al(III)-FLU complex.**

**Figure 3:** Optimized structure of (a) flubendazole and (b) flubendazole -Al(III) complex.

Table 1. Some of the optimized bond lengths, Å and bond angles, degrees, for FLU and its complexe using B3LYP/6-311G and B3LYP/LANL2DZ respectively.

| Bond length (A^o^) | FLU | Al-complex |
| --- | --- | --- |
| R(C6-C8) | 1.49484 | 1.47650 |
| R(C8-O9) | 1.25378 | 1.25734 |
| R(C8-C10) | 1.48653 | 1.52634 |
| R(C13-N16) | 1.38185 | 1.43170 |
| R(N16-C18) | 1.32558 | 1.37228 |
| R(C17-C18) | 1.37731 | 1.35196 |
| R(C18-N19) | 1.38744 | 1.40601 |
| R(N19-C20) | 1.37020 | 1.37925 |
| R(C20-O21) | 1.24384 | 1.29367 |
| R(C20-O22) | 1.36833 | 1.29972 |
| R(O22-C23) | 1.47157 | 1.46800 |
| R(N16-Al) | --- | 1.88515 |
| R(O21-Al) | --- | 1.79538 |
| Bond angles, degrees | | |
| A(C23-O22-C20) | 116.690 | 127.608 |
| A(O22-C20-O21) | 124.690 | 118.629 |
| A(O22-C20-N19) | 110.207 | 123.176 |
| A(N19-C20-O21) | 125.103 | 118.195 |
| A(C20-N19-C18) | 124.936 | 123.575 |
| A(N19-C18-N16) | 122.955 | 125.992 |
| A(N19-C18-N17) | 122.980 | 123.143 |
| A(C18-N16-Al) | --- | 123.184 |
| A(C13-N16-Al) | --- | 130.466 |
| A(C14-C13-N16) | 129.908 | 131.126 |

E, mV

| -log[Al(III)] | Al |
| --- | --- |
| 7 | 166 |
| 6 | 194 |
| 5 | 213 |
| 4 | 228 |
| 3 | 245 |
| 2 | 270 |
| 1.3 | 284 |
| 1 | 291 |

E, mV

| -log [Mn+] | cu | ca | cr | Ba | sr | co | Ni | Mn | mg | pb | Na | K | Al |
| --- | --- | --- | --- | --- | --- | --- | --- | --- | --- | --- | --- | --- | --- |
| 7 | 156 | 194 | 166 | 144 | 158 | 102 | 121 | 130 | 130 | 54 | 71 | 51 | 166 |
| 6 | 219 | 186 | 189 | 151 | 170 | 94 | 106 | 133 | 142 | 55 | 44 | 31 | 194 |
| 5 | 227 | 183 | 178 | 148 | 171 | 105 | 88 | 151 | 144 | 90 | 80 | 54 | 213 |
| 4 | 231 | 196 | 191 | 163 | 176 | 116 | 100 | 170 | 159 | 109 | 88 | 91 | 228 |
| 3 | 244 | 211 | 208 | 191 | 201 | 133 | 128 | 181 | 188 | 110 | 102 | 100 | 245 |
| 2 | 274 | 230 | 226 | 216 | 222 | 148 | 150 | 192 | 206 | 102 | 135 | 136 | 270 |
| 1.3 | 292 | 246 | 233 | 233 | 188 | 166 | 168 | 179 | 202 | 105 | 152 | 156 | 284 |
| 1 | 322 | 254 | 242 | 231 | 163 | 178 | 180 | 181 | 196 | 108 | 171 |  | 291 |

**Figure 4: calibration curve of Al(III) ion solution using sensor IV.**

**Figure 5: calibration plots of different metal ion using sensor IV.**

|  |  |
| --- | --- |
| 2 | 274 |
| 2.2 | 274 |
| 2.4 | 272 |
| 2.6 | 269 |
| 2.8 | 258 |
| 3 | 252 |
| 3.2 | 241 |
| 3.4 | 230 |
| 3.8 | 226 |
| 4.2 | 222 |
| 4.5 | 222 |
| 4.7 | 223 |
| 5 | 225 |
| 5.2 | 225 |
| 5.5 | 225 |
| 6 | 215 |
| 7 | 180 |

**Figure 6: pH as a function of potential of the sensor IV.**
